# Trificial Reflexes with Special Reference to Diseases of the Eye, Ear and Nose

**Published:** 1913-02

**Authors:** 


TRIFICIAL REFLEXES WITH SPECIAL REFERENCE TO DISEASES OF THE EYE, EAR AND NOSE.—Albert H. Andrews of Chicago (*Journal Oph. and Oto.-Laryng.*, September, 1912), discusses three phases of the subject: the anatomic, the physiologic and the clinical.

Assuming that an irritation of the terminal filaments of a sensory nerve may produce a disturbance of sensation referred to the terminal filaments of another branch of the same nerve, it is easy to see how disturbance of vision through the ciliary nerves, lenticular ganglion, and the cavernous branch may produce frontal headaches; or through the Gasserian ganglion to the branch of the tentorium cerebelli may produce occipital headaches. It is also easy to see how in disease or malformation of the turbinate bodies making pressure on the septum, the pain may be referred to the eyes. When we remember that the iris and ciliary body are supplied by branches of this nerve, we can understand why pain in the eye of nasal origin may be increased by using the eye for close work, even when there is no refractive error. Non-suppurative diseases produce reflex disturbances, while suppurative diseases do not because in the latter the suppurative processes destroy the sensory nerve terminals.

He enumerates a dozen cases, each one illustrating a different reflex disturbance through the distribution of the trifacial—pain in the ear and headache from antrum disease—antrum disease affecting the teeth; pain in the ear from dental disease; turbinate trouble causing eye distress; disease of the turbinals causing headache and vomiting; ethmoiditis causing eye trouble and neurasthenia; cough produced by atrophic rhinitis, also by atrophic tonsil, etc.

